# Dependence of cognitive ability on synchronous neural interactions determined by magnetoencephalography

**DOI:** 10.1152/jn.00077.2023

**Published:** 2023-03-29

**Authors:** Lisa M. James, Arthur C. Leuthold, Stacy Dolan, Apostolos P. Georgopoulos

**Affiliations:** ^1^The Cognitive Neuroscience Research Group, Brain Sciences Center, Minneapolis Veterans Affairs Health Care System, Minneapolis, Minnesota, United States; ^2^Department of Neuroscience, University of Minnesota Medical School, Minneapolis, Minnesota, United States; ^3^Department of Psychiatry, University of Minnesota Medical School, Minneapolis, Minnesota, United States; ^4^Center for Cognitive Sciences, University of Minnesota, Minneapolis, Minnesota, United States; ^5^Department of Neurology, University of Minnesota Medical School, Minneapolis, Minnesota, United States

**Keywords:** cognition, decorrelation, magnetoencephalography

## Abstract

Previous studies have shown that synchronous neural interactions (SNIs) underlying healthy brain function can be readily distinguished from neural anomalies associated with diseases including dementia; however, it is imperative to identify biomarkers that facilitate early identification of individuals at risk for cognitive decline before the onset of clinical symptoms. Here, we evaluated whether variation in brain function, controlling for age, corresponds with subtle decrements in cognitive performance in cognitively healthy women. A total of 251 women (age range 24–102 yr) who performed above established cutoffs on the Montreal cognitive assessment (MoCA) also underwent a task-free magnetoencephalography scan from which SNIs were computed. The results demonstrated that increased SNI was significantly associated with decreased cognitive performance (*r*^2^ = 0.923, *P* = 0.009), controlling for age. Compared with the lowest performers with normal cognition (MoCA = 26), SNI of the highest performers (MoCA = 30) was associated with decorrelation primarily in the right anterior temporal cortex region, with additional (weaker) foci in left anterior temporal cortex, right posterior temporal cortex, and cerebellum. The findings highlight the relevance of neural network decorrelation on cognitive functioning and suggest that subtle increases in SNI may presage future cognitive impairment.

**NEW & NOTEWORTHY** This study in cognitively healthy women showed that decreased cognitive performance is associated with increased neural network correlations, particularly involving the temporal cortices. As healthy brain function relies on dynamic neural network communication, these findings suggest that subtle increases in correlated neural network activity may be a useful early indicator of decrements in cognitive function.

## Graphical abstract

**Figure:**
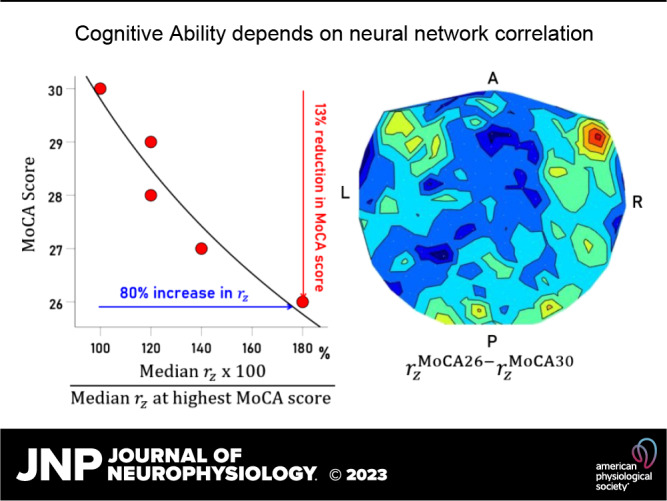

## INTRODUCTION

Healthy brain function relies on communication between neuronal populations in a vast, interconnected neural network. Variation in the dynamic nature of neural activity, which can be captured with magnetoencephalography (MEG), provides a useful biomarker of brain disorders (1). Prior MEG studies have shown that patterns of at-rest neural network activity are remarkably similar and synchronous across healthy individuals (2), so much so that signature deviations in synchronous neural interactions (SNIs) reflect specific types of brain pathology (1, 3–6). Moreover, disease progression and recovery are reflected in variation in brain function assessed with MEG (7–10). Several MEG studies have evaluated brain function in Alzheimer’s dementia, a condition characterized by loss of cognitive capabilities. Findings from those studies have pointed to loss of functional connectivity (11) and increased SNI in patients with dementia compared with healthy controls (10). Subtle brain changes are evident long before the onset of clinical symptoms (12, 13); therefore, early detection of those changes may facilitate identification of individuals at risk for cognitive decline. Here, in a sample of cognitively healthy women, we evaluated whether variation in brain function corresponds with variation in scores reflecting normal cognitive functioning on the Montreal cognitive assessment (MoCA, 14), a well-validated cognitive screening measure. Since SNI changes with age, particularly in genetically vulnerable individuals (15), age was controlled for in the present analyses to ascertain the association of cognitive ability with SNI in the absence of age effects.

## MATERIALS AND METHODS

### Participants

**Figure 1.**
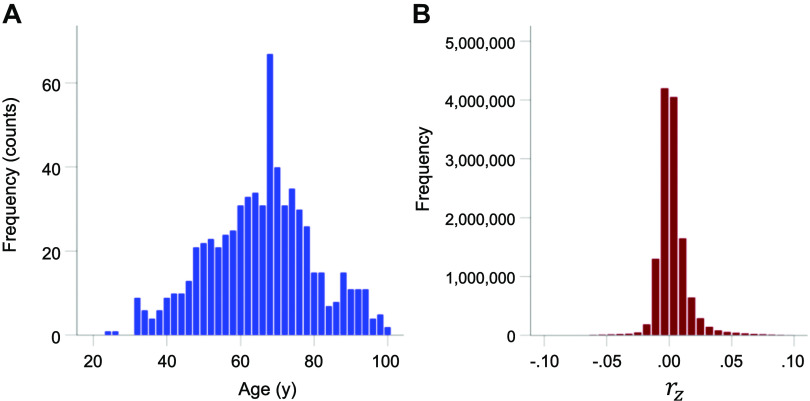
*A*: frequency distribution of age of participants at the time of data acquisition (*n* = 667 participants). *B*: frequency distribution of *r_z_* (*n* = 13,244,413 cross-correlations).

A total of 251 cognitively healthy women participated in this study as paid volunteers after providing written informed consent, in adherence to the Declaration of Helsinki; all study protocols were approved by the appropriate Institutional Review Boards. Data were acquired at repeated visits (one or more years apart) for a number of participants, for a total of 667 visits. The mean age of participants at the time of acquisition (visit) was 65.06 ± 14.47 yr (means ± SD, age range 24–102 yr; Fig. 1*A*). Women were excluded from participation if they had been diagnosed with any condition affecting brain function including neurological disorders, psychiatric disorders, autoimmune conditions with prominent neurocognitive symptoms, history of brain trauma or injury, brain cancer, and any other cancers requiring radiation or chemotherapy due to potential effects on the brain.

The cognitive status of all participants at the time of data acquisition was assessed using the MoCA (14) and scores for all participants were >25, a commonly used threshold indicative of healthy cognitive functioning (range 25–30; no extra point was added for education). The percentages of the various MoCA scores were as follows: MoCA 26 (14.2%), 27 (22.3%), 28 (22.0%), 29 (23.8%), and 30 (17.5%).

### MEG Data Acquisition

All participants underwent a MEG scan. As described previously (1), subjects lay supine within the electromagnetically shielded chamber and fixated their eyes on a spot 65 cm in front of them, for 60 s. MEG data were acquired using a 248-channel axial gradiometer system (Magnes 3600WH, 4-D Neuroimaging, San Diego, CA), band-filtered between 0.1 and 400 Hz, and sampled at 1,017.25 Hz, corresponding to a sampling interval of 0.983 ms (rounded to 1 ms for convenience in conveying the results detailed later). Data with artifacts (e.g., from excessive subject motion) were eliminated from further analysis. MEG records were visually screened and rejected if artifacts were present (e.g., from excessive subject motion, eye movements, blinking, or environmental noise).

### MEG Data Processing

Processing of the raw MEG series was performed using programs in Python (16). Single-trial MEG time series from all sensors underwent “prewhitening” (17) using a (50, 1, 3) ARIMA model to obtain innovations (i.e., residuals) (16). All possible pairwise zero-lag crosscorrelations, *r* (synchronous neural interactions, SNIs; *n* = $\frac{248\;x\;247}{2}$ = 30,628 sensor pairs) were computed between the prewhitened MEG time series of each MEG scan. Crosscorrelations were transformed to *r_z_* using Fisher’s (18) z-transformation to normalize their distribution:

(*1*) $$\text{SNI}:\;r_{z}=\text{arctanh}\left(r\right)=\frac{1}{2}\ln\left(\frac{1+r}{1-r}\right)$$

### Data Analysis

An analysis of covariance (ANCOVA) was used to evaluate the effect of MoCA on *r_z_* (with age as covariate) and a regression analysis to investigate the nature of the possible dependence of MoCA on *r_z_*. Nonparametric statistics included calculation and comparison of median *r_z_* for the five MoCA groups. Statistical analyses were performed using the IBM-SPSS statistical package (v.27). All *P* values reported are two-tailed. Brain area location was tentatively identified using the BESA (Gräfelfing, Germany) and Brain Voyageur (Maastricht, The Netherlands) software.

## RESULTS

### General

**Figure 2.**
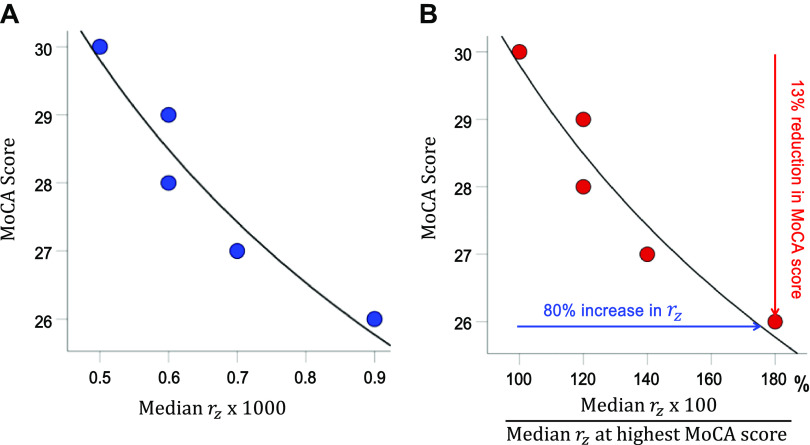
*A*: association of Montreal cognitive assessment (MoCA) score and median *r_z_*. *B*: plot of the MoCA score against *r_z_* standardized to the best MoCA score of 30. See text for details.

The frequency distribution of *r_z_* is shown in Fig. 1*B.* An ANCOVA revealed a highly statistically significant effect of MoCA (*F* test, *P* < 0.001; also independent-samples median test, *P* < 0.001). More specifically, MoCA score decreased with *r_z_* as a power fit (Fig. 2*A*); the fit was excellent (*r*^2^ = 0.923, *P* = 0.009, *n* = 5). Figure 2*B* depicts the same information as in Fig. 2*A* but on a *r_z_* scale standardized to the best MoCA score of 30. It can be seen that an increase in *r_z_* by 80% was associated with a reduction in the MoCA score by 13%.

### Brain Representation

**Figure 3.**
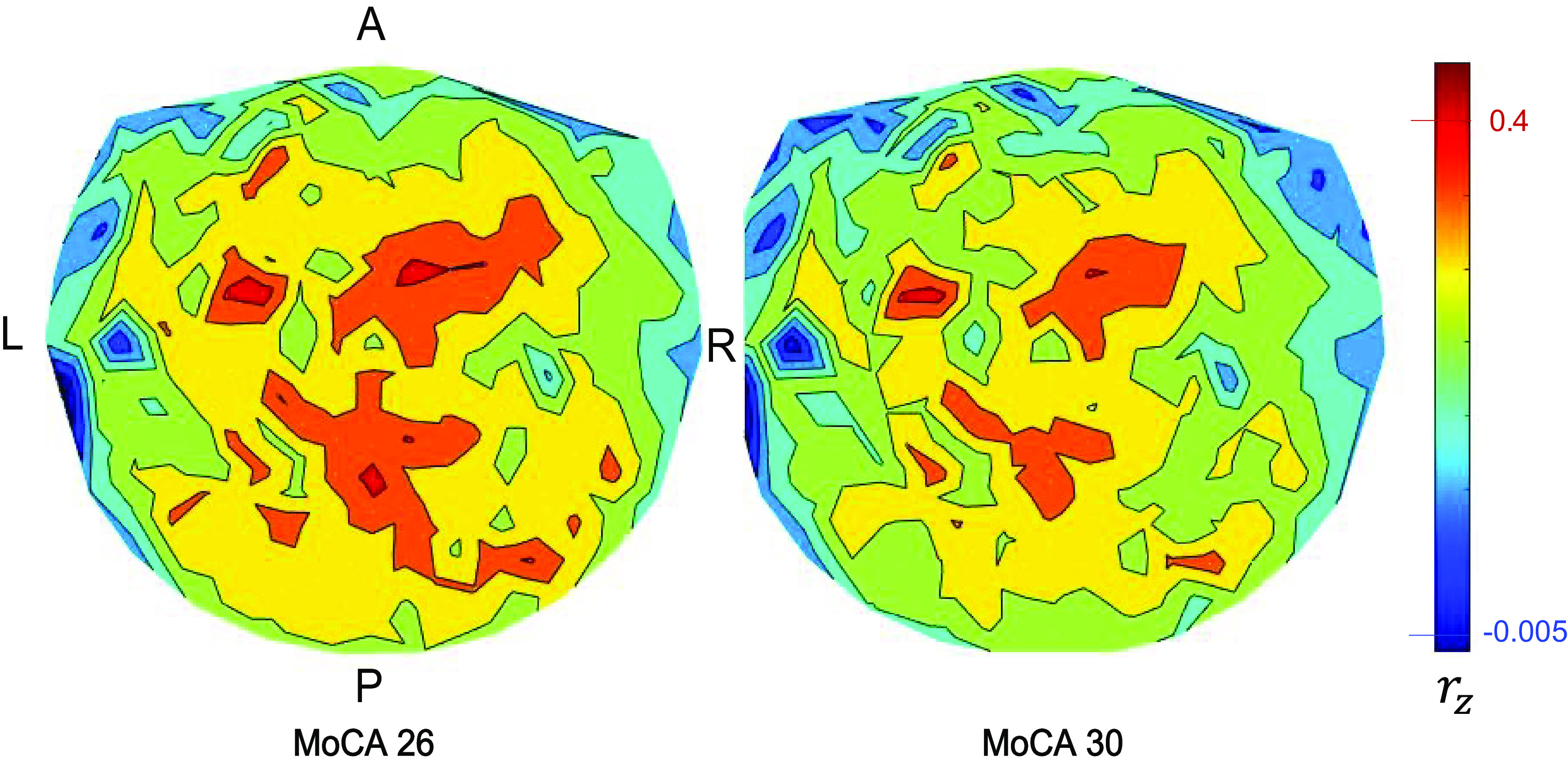
Heatmaps of *r_z_* [averaged per magnetoencephalography (MEG) sensor] for the lowest and highest Montreal cognitive assessment (MoCA) score (26 and 30, respectively). A, anterior; L, left; P, posterior; R, right. See text for details.

**Figure 4.**
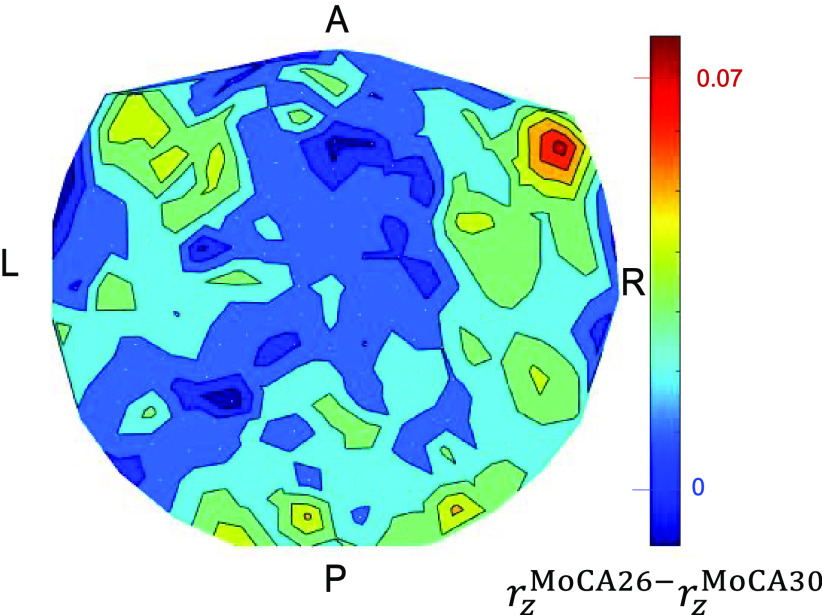
Heatmap of the difference between the Montreal cognitive assessment (MoCA) 26 – MoCA 30 heatmaps of Fig. 3. See text for details. A, anterior; L, left; P, posterior; R, right.

We visualized the distribution in the brain of the interactions *r_z_* involved in the reduction of *r_z_* with better performance in the task (higher MoCA scores) by computing the mean *r_z_* for each sensor with the rest 247 sensors for each MoCA score (26–30), and plotting it in a brain MEG-sensor heatmap. Figure 3 shows two such heatmaps in sensor space, namely, one for the lowest MoCA score of 26 (Fig. 3*A*) and the other for the highest MoCA score of 30 (Fig. 3*B*). It can be seen that the higher MoCA score of 30 comprised weaker *r_z_*, with respect to their spread and intensity. This is further illustrated in Fig. 4 that plots the difference of *r_z_* of MoCA-26 minus that of MoCA 30. It can be seen that the source of the highest decorrelation in MoCA-30 *r_z_* was in the right anterior temporal cortex region, with additional (weaker) foci in left anterior temporal cortex (mirror of the right), right posterior temporal cortex, and cerebellum.

## DISCUSSION

Here, we evaluated neural network communication, the essence of healthy brain functioning, at different cognitive performance scores, all of which were indicative of normal cognitive functioning according to a widely used screening assessment. The findings documented that even among cognitively normal women, brain function varied systematically with cognitive performance. Specifically, as cognitive performance decreased from optimal performance and approached a cutoff score indicative of possible cognitive impairment, cross-correlated neural activity *r_z_* (i.e., SNI) increased. MoCA performance has been shown to predict conversion from mild cognitive impairment to dementia (19), and increased SNI has been documented in individuals with Alzheimer’s dementia compared with controls (10). The current findings suggest that subtle increases in SNI even among cognitively healthy individuals may portend future cognitive impairment.

Healthy cognitive functioning is predicated on dynamic neural network communication. Here, we showed that subtle decrements in cognitive performance were associated with an increasingly correlated neural network. Previous research has highlighted the importance of decorrelation in information processing (20, 21). In essence, decorrelation is a strategy observed across systems (e.g., visual, motor) and species that enhances information encoding, storage, and retrieval (20). Our results showed that as the neural network became more constrained (indicated by increased SNI), decrements in cognitive processing were observed and, conversely, relatively decorrelated networks supported enhanced cognitive performance. Thus, the present findings speak to the importance of neural network decorrelation as a mechanism that extends beyond sensory and motor systems to facilitate normal cognitive functioning. Of note, both hyper- and hypo-correlated neural activity are associated with pathology (6, 22), suggesting that normal cognition may lie within certain bounds of optimal neural network correlation, bounds that remain to be determined.

Notably, differences in SNI between the highest and lowest performers within this cognitively normal group were relatively localized. The largest difference was observed in the right anterior temporal cortex with weaker effects observed in the left anterior temporal cortex, right posterior temporal cortex, and cerebellum. The anterior temporal cortices are connected with several brain areas and are consequently implicated in diverse functions including semantic learning and memory and social cognition (23). Notably, anterior temporal lobe anomalies have been linked to a subtype of frontotemporal dementia known as semantic dementia that is characterized by prominent semantic deficits as reviewed elsewhere (23). Among patients with semantic dementia, hallmark semantic errors have been associated with right temporal lobe atrophy, especially in posterior temporal areas, and deficits in naming and comprehension have been associated with left anterior and posterior temporal atrophy, respectively (24). In patients with frontotemporal dementia, right anterior temporal lobe changes have been linked to greater social and emotional impairment than left anterior temporal lobe involvement (25, 26). Furthermore, the right anterior temporal lobe has been implicated in re-experiencing sensory phenomena upon stimulation (27) and de novo in individuals with posttraumatic stress disorder (7, 22). Although social, emotional, and semantic deficits have been linked to anterior and posterior temporal anomalies in patient populations, it is unclear whether our findings of temporal anomalies in a healthy population are a harbinger of future deficits akin to those observed in frontotemporal or other types of dementia.

## DATA AVAILABILITY

Data will be made available upon reasonable request.

## GRANTS

Partial funding for this study was provided by the University of Minnesota (the Anita Kunin Chair in Women’s Healthy Brain Aging, the Brain and Genomics Fund McKnight Presidential Chair of Cognitive Neuroscience, the American Legion Brain Sciences Chair) and the US Department of Veterans Affairs.

## DISCLAIMERS

The sponsors had no role in the current study design, analysis or interpretation, or in the writing of this paper. The contents do not represent the views of the US Department of Veterans Affairs or the US Government.

## DISCLOSURES

No conflicts of interest, financial or otherwise, are declared by the authors.

## AUTHOR CONTRIBUTIONS

A.P.G. conceived and designed research; L.M.J., A.C.L., and S.D. performed experiments; A.P.G. analyzed data; L.M.J., A.C.L., and A.P.G. interpreted results of experiments; A.P.G. prepared figures; L.M.J. and A.P.G. drafted manuscript; L.M.J., A.C.L., S.D., and A.P.G. edited and revised manuscript; L.M.J., A.C.L., S.D., and A.P.G. approved final version of manuscript.

## References

[1] Georgopoulos AP, Karageorgiou E, Leuthold AC, Lewis SM, Lynch JK, Alonso AA, Aslam Z, Carpenter AF, Georgopoulos A, Hemmy LS, Koutlas IG, Langheim FJP, McCarten JR, McPherson SE, Pardo JV, Pardo PJ, Parry GJ, Rottunda SJ, Segal BM, Sponheim SR, Stanwyck JJ, Stephane M, Westermeyer JJ. Synchronous neural interactions assessed by magnetoencephalography: a functional biomarker for brain disorders. J Neural Eng 4: 349–355, 2007. doi:10.1088/1741-2560/4/4/001. 18057502

[2] Langheim FJ, Leuthold AC, Georgopoulos AP. Synchronous dynamic brain networks revealed by magnetoencephalography. Proc Natl Acad Sci USA 103: 455–459, 2006. doi:10.1073/pnas.0509623102. 16387850 PMC1324790

[3] Georgopoulos AP, Tan HM, Lewis SM, Leuthold AC, Winskowski AM, Lynch JK, Engdahl B. The synchronous neural interactions test as a functional neuromarker for post-traumatic stress disorder (PTSD): a robust classification method based on the bootstrap. J Neural Eng 7: 016011, 2010. doi:10.1088/1741-2560/7/1/016011. 20086271

[4] Engdahl BE, James LM, Miller RD, Leuthold AC, Lewis SM, Carpenter AF, Georgopoulos AP. A magnetoencephalographic (MEG) study of Gulf War Illness (GWI). EBioMedicine 12: 127–132, 2016. doi:10.1016/j.ebiom.2016.08.030. 27592598 PMC5078573

[5] James LM, Leuthold AF, Georgopoulos AP. MEG neural signature of sexual trauma in women veterans with PTSD. Exp Brain Res 240: 2135–2142, 2022. doi:10.1007/s00221-022-06405-8. 35786746

[6] Voytek B, Knight RT. Dynamic network communication as a unifying neural basis for cognition, development, aging, and disease. Biol Psychiatry 77: 1089–1097, 2015. doi:10.1016/j.biopsych.2015.04.016. 26005114 PMC4443259

[7] Engdahl B, Leuthold AC, Tan HM, Lewis SM, Winskowski AM, Dikel TN, Georgopoulos AP. Post-traumatic stress disorder: a right temporal lobe syndrome? J Neural Eng 7: 066005, 2010. doi:10.1088/1741-2560/7/6/066005. 20980718

[8] Thorpe DR, Engdahl BE, Leuthold A, Georgopoulos AP. Assessing recovery from mild traumatic brain injury (mtbi) using magnetoencephalography (MEG): an application of the synchronous neural interactions (SNI) test. J Neurol Neuromed 5: 28–34, 2020. doi:10.29245/2572.942X/2020/3.1274. PMC12311912 40746876

[9] James LM, Leuthold AF, Georgopoulos AP. Classification of posttraumatic stress disorder and related outcomes in women veterans using magnetoencephalography. Exp Brain Res 240: 1117–1125, 2022. doi:10.1007/s00221-022-06320-y. 35133447

[10] Verdoorn TA, McCarten JR, Arciniegas DB, Golden R, Moldauer L, Georgopoulos A, Lewis S, Cassano M, Hemmy L, Orr W, Rojas DC. Evaluation and tracking of Alzheimer’s disease severity using resting-state magnetoencephalography. J Alzheimers Dis 26: 239–255, 2011. doi:10.3233/JAD-2011-0056. 21971464

[11] Mandal PK, Banerjee A, Tripathi M, Sharma A. A comprehensive review of magnetoencephalography (MEG) studies for brain functionality in healthy aging and Alzheimer’s disease (AD). Front Comput Neurosci 12: 60, 2018. doi:10.3389/fncom.2018.00060. 30190674 PMC6115612

[12] Ewers M, Sperling RA, Klunk WE, Weiner MV, Hampel H. Neuroimaging markers for the prediction and early diagnosis of Alzheimer’s disease dementia. Trends Neurosci 34: 430–442, 2011. doi:10.1016/j.tins.2011.05.005. 21696834 PMC3275347

[13] Davatzikos C, Xu F, An Y, Fan Y, Resnick SM. Longitudinal progression of Alzheimer’s-like patterns of atrophy in normal older adults: the SPARE-AD index. Brain 132: 2026–2035, 2009. doi:10.1093/brain/awp091. 19416949 PMC2714059

[14] Nasreddine ZS, Phillips NA, Bédirian V, Charbonneau S, Whitehead V, Collin I, Cummings JL, Chertkow H. The Montreal Cognitive Assessment, MoCA: a brief screening tool for mild cognitive impairment. J Am Geriatr Soc 53: 695–699, 2005. doi:10.1111/j.1532-5415.2005.53221.x. 15817019

[15] James LM, Dolan S, Leuthold AC, Engdahl BE, Georgopoulos A, Georgopoulos AP. The effects of human leukocyte antigen DRB1* 13 and apolipoprotein E on age-related variability of synchronous neural interactions in healthy women. EBioMedicine. 35: 288–294, 2018. doi:10.1016/j.ebiom.2018.08.026. 30139626 PMC6161538

[16] Mahan MY, Leuthold AC, Georgopoulos AP. Spatiotemporal brain network analysis of healthy humans based on magnetoencephalography and functional MRI in the resting state. BMC Neurosci 16: 155, 2015. doi:10.1186/1471-2202-16-S1-P155.

[17] Box GE, Jenkins GM, Reinsel GC, Ljung GM. Time Series Analysis: Forecasting and Control. Hoboken, NJ: Wiley, 2015.

[18] Fisher RA. Statistical Methods for Research Workers (13th ed.). Edinburgh: Oliver & Boyd, 1958.

[19] Julayanont P, Brousseau M, Chertkow H, Phillips N, Nasreddine ZS. Montreal Cognitive Assessment Memory Index Score (MoCA‐MIS) as a predictor of conversion from mild cognitive impairment to Alzheimer's Disease. J Am Geriat Soc 62: 679–684, 2014. doi:10.1111/jgs.12742. 24635004

[20] Olshausen BA, Field DJ. Sparse coding of sensory inputs. Curr Opin Neurobiol 14: 481–487, 2004. doi:10.1016/j.conb.2004.07.007. 15321069

[21] Vinje WE, Gallant JL. Sparse coding and decorrelation in primary visual cortex during natural vision. Science 287: 1273–1276, 2000. doi:10.1126/science.287.5456.1273. 10678835

[22] James LM, Engdahl BE, Leuthold AC, Lewis SM, Van Kampen E, Georgopoulos AP. Neural network modulation by trauma as a marker of resilience: differences between veterans with posttraumatic stress disorder and resilient controls. JAMA Psychiat 70: 410–418, 2013. doi:10.1001/jamapsychiatry.2013.878. 23426853

[23] Wong C, Gallate J. The function of the anterior temporal lobe: a review of the empirical evidence. Brain Res 1449: 94–116, 2012. doi:10.1016/j.brainres.2012.02.017. 22421014

[24] Snowden JS, Harris JM, Thompson JC, Kobylecki C, Jones M, Richardson AM, Neary D. Semantic dementia and the left and right temporal lobes. Cortex 107: 188–203, 2018. doi:10.1016/j.cortex.2017.08.024. 28947063

[25] Edwards-Lee T, Miller BL, Benson DF, Cummings JL, Russell GL, Boone K, Mena I. The temporal variant of frontotemporal dementia. Brain 120: 1027–1040, 1997. doi:10.1093/brain/120.6.1027. 9217686

[26] Thompson SA, Patterson K, Hodges JR. Left/right asymmetry of atrophy in semantic dementia Behavioral–cognitive implications. Neurology 61: 1196–1203, 2003. doi:10.1212/01.WNL.0000091868.28557.B8. 14610120

[27] Gloor P. Experiential phenomena of temporal lobe epilepsy. Facts and hypotheses. Brain 113: 1673–1694, 1990. doi:10.1093/brain/113.6.1673. 2276040

